# Controversies in enhanced recovery after cardiac surgery

**DOI:** 10.1186/s13741-022-00250-7

**Published:** 2022-04-28

**Authors:** Andrew D. Shaw, Nicole R. Guinn, Jessica K. Brown, Rakesh C. Arora, Kevin W. Lobdell, Michael C. Grant, Tong J. Gan, Daniel T. Engelman, for the Perioperative Quality Initiative (POQI) and Enhanced Recovery after Surgery–Cardiac (ERAS→-Cardiac) investigators

**Affiliations:** 1 Department of Intensive Care and Resuscitation, Cleveland Clinic, 9500 Euclid Avenue, Cleveland, OH 44195 USA; 2 Department of Anesthesiology, Duke University Medical Center, Box 3094, 2301 Erwin Road, Durham, NC USA; 3 Department of Anesthesiology and Perioperative Medicine, Division of Anesthesiology and Critical Care, The University of Texas MD Anderson Cancer Center, Houston, TX USA; 4 Cardiac Surgery, Department of Surgery, Max Rady College of Medicine, University of Manitoba, Winnipeg, MB Canada; 5 Education and Research, Atrium Health, Charlotte, NC USA; 6 Departments of Anesthesiology/Critical Care Medicine and Surgery, The Johns Hopkins Medical Institutions, 1800 Orleans Street, Baltimore, MD USA; 7 Department of Anesthesiology, Stony Brook University Renaissance School of Medicine, Stony Brook, NY USA; 8 University of Massachusetts Medical School-Baystate, Baystate Medical Center, 759 Chestnut St, Springfield, MA USA

**Keywords:** Enhanced recovery after surgery, Patient blood management, Acute kidney injury, Opioid analgesia reduction, Delirium

## Abstract

Advances in cardiac surgical operative techniques and myocardial protection have dramatically improved outcomes in the past two decades. An unfortunate and unintended consequence is that 80% of the preventable morbidity and mortality following cardiac surgery now originates outside of the operating room. Our hope is that a renewed emphasis on evidence-based best practice and standardized perioperative care will reduce overall morbidity and mortality and improve patient-centric care. The Perioperative Quality Initiative (POQI) and Enhanced Recovery After Surgery–Cardiac Society (ERAS® Cardiac) have identified significant evidence gaps in perioperative medicine related to cardiac surgery, defined as areas in which there is significant controversy about how best to manage patients. These five areas of focus include patient blood management, goal-directed therapy, acute kidney injury, opioid analgesic reduction, and delirium.

**Supplementary Information:**

The online version contains supplementary material available at 10.1186/s13741-022-00250-7.

## Background

Enhanced Recovery After Surgery (ERAS) programs have become ubiquitous in the field of perioperative medicine and have played a key role in defining the essence of this relatively young discipline. These protocols have typically been applied to non-cardiac surgical populations (Wennström et al. 2020; Smith et al. 2019; Gupta and Senagore 2017), but more recently they have also been applied to cardiac surgery patients (Engelman et al. 2019). While consensus has emerged in some areas of perioperative management for patients undergoing heart surgery, considerable differences of opinion remain for others. This reflects a relative lack of evidence in the field and has led to differences in treatment plans for patients undergoing the same procedure, usually in different centers but sometimes even within the same institution.

The Perioperative Quality Initiative (POQI) and Enhanced Recovery After Surgery–Cardiac Society (ERAS→-Cardiac) organizations are both committed to excellence in perioperative medicine, and in particular for patients undergoing high-risk surgery (Shannon et al. 2012). Recognizing that best practice comes from research addressing genuine evidence gaps, these two groups have developed this brief review of important topics in perioperative medicine related to cardiac surgery. The specific goal of this paper therefore is to review those areas of high importance, themselves defined as areas in which there is significant controversy about how best to manage patients. We have focused on five topics—those in which we believe there are answerable questions that address specific problems faced by cardiac surgical patients, and in which well-designed research studies will advance the field by providing specific answers. These topics are patient blood management, goal-directed therapy, acute kidney injury, opioid analgesic reduction, and delirium. We address each topic individually, and provide a list of focused research questions (Table 1) which we believe represent high priority areas for perioperative cardiac researchers to address.

**Table 1:** Focused research questions

Topic area	***Priority 1***	***Priority 2***	***Priority 3***
**Patient blood management**	*How and when should patients undergoing non-emergent cardiac surgery be evaluated and treated for preoperative anemia?*	*What is the appropriate hemoglobin concentration to trigger transfusion before, during, and after cardiac surgery?*	*What is the role of acute normovolemic hemodilution and autologous pump priming in patient blood management during cardiac surgery?*
**Goal-directed hemodynamic therapy**	*What is the mechanism by which goal-directed therapy improves outcomes in cardiac surgical patients?*	*Which elements of GDT have the greatest impact on outcomes?*	*Which aspects of GDT are specific to cardiac surgical patients?*
**Acute kidney injury**	*How do novel biomarkers contribute to the prediction of postoperative AKI?*	*What aspects of kidney replacement therapy should be individualized to the specific patient—is there a role for goal-directed dialysis?*	*How do anesthetic pharmacologic strategies impact the incidence of CSA-AKI?*
**Opioid reduction**	*Which non-opioid medications and techniques provide optimal analgesia in the cardiac surgical population?*	*How do opioid-sparing strategies contribute to a meaningful reduction in opioid use after cardiac surgery?*	*How does chronic opioid use impact perioperative pain management in the cardiac surgical setting?*
**Delirium**	*What is the role of specific sedative drugs in the development, prevention, and management of delirium in the postoperative cardiac surgery patient?*	*What is the most valid and effective delirium screening tool in the post-operative cardiac surgery ward?*	*What non-drug treatments might reduce the incidence of postoperative delirium?*

## Main text

### Patient blood management (PBM)

With an increased focus on providing value-based care by improving patient outcomes and decreasing costs, attention has turned to the role of patient blood management (PBM) to reduce unnecessary transfusions for patients undergoing cardiac surgery (Spahn et al. 2020). While many PBM methods have become widely adopted to reduce red blood cell transfusion in cardiac surgery, including routine use of anti-fibrinolytics (Derzon et al. 2019), restrictive transfusion thresholds (Hajjar et al. 2010; Mazer et al. 2017) and cell salvage (Carless et al. 2010), there remain some areas of controversy regarding other safe and effective PBM techniques.

A major area of debate in the perioperative management of cardiac surgery patients encompasses preoperative anemia. Although there is a growing body of evidence demonstrating that treating patients with iron deficiency or anemia prior to non-emergent cardiac surgery reduces transfusions (Weltert et al. 2015; Yoo et al. 2011; Spahn et al. 2019), we still lack consensus on how to screen patients for anemia, and who, how, and when to treat (Task Force on Patient Blood Management for Adult Cardiac Surgery of the European Association for Cardio-Thoracic, Surgery, the European Association of Cardiothoracic, Anaesthesiology, and Boer 2018). The ideal hemoglobin target has not been determined, and it remains unknown whether treatment thresholds should differ for men and women. The World Health Organization definition of anemia is a hemoglobin concentration of less than 12 g/dL in women and 13 g/dL in men. Treatment may include iron alone (intravenous or oral) or in combination with erythropoietin stimulating agents (ESAs), but the ideal dosing and timing of treatment has yet to be determined. Due to the urgent nature of many cardiac surgeries, recent studies have focused on a short preoperative course, with anemia treated only days prior to surgery (Weltert et al. 2015; Yoo et al. 2011; Spahn et al. 2019), although a full response to treatment would be expected to take considerably longer. In addition to the higher costs in comparison to IV iron alone, use of ESAs in cardiac and vascular surgery patients remains off-label in the USA due to the Food and Drug Administration’s Black Box warning of potential increased risk of thromboembolism. The patients who would most benefit from the decreased transfusion risks without significantly increasing thromboembolism risk remain uncertain.

Other areas of controversy include transfusion triggers while on cardiopulmonary bypass, intraoperatively, before and after revascularization, and postoperatively (Ferraris et al. 2011; Murphy et al. 2015). Despite evidence supporting equivalent outcomes with restrictive versus liberal transfusion triggers (Hajjar et al. 2010), the relative risk of ischemia related to anemia for each of these timeframes remains unknown. Understandably, the decision to transfuse involves many factors, including patient age and comorbidities, baseline hemoglobin, and level of bleeding. But guidelines, both in terms of hemoglobin triggers and thresholds for patients undergoing cardiac surgery with cardiopulmonary bypass remain controversial.

Finally, although cell salvage has become a well-established non-pharmacologic technique to reduce transfusion in cardiac surgery patients, other techniques, including acute normovolemic hemodilution (Zhou et al. 2015) and autologous pump priming (Saczkowski et al. 2009; Hou et al. 2009; Vandewiele et al. 2013) are less widely employed and the literature is mixed. Although each method tends to show a reduction in total red blood cell transfusion, controversy remains regarding which patients are most likely to benefit from these techniques. Further, their use can be challenging in patients with lower circulating blood volume who may require relative euvolemia as well as an adequate hemoglobin concentration.

### Goal-directed therapy (GDT)

The fundamental underlying principle of goal-directed therapy (GDT) relates to matching therapy with patient demands and avoiding the outstripping of the patient’s reserve throughout their hospital course. Clinical best practice includes quantitative risk assessment (Society of Thoracic Surgeons 2021; Nashef et al. 2002) when feasible and mitigation of modifiable risks. Controversy surrounding the merits of GDT may be pragmatically categorized by the phase(s) of care and related to process issues such as monitoring, therapeutics, personnel, safety, and cost.

As a result of monitoring capabilities and patient specific risks, the most common applicable phases of care for GDT in cardiac surgery include the operating room (OR) and intensive care unit (ICU) (O’Neal and Shaw 2016). Within the OR, one must consider both anesthetic methods—which increasingly include locoregional analgesia and less invasive monitoring—and perfusion practices. Both of these methods must be tailored to the surgical procedure and specific techniques, i.e., sternotomy, thoracotomy, CABG, OPCAB, valve replacement, and/or minimally invasive incisions.

The specific elements of GDT (Kapoor et al. 2017; Cheng et al. 2020) may be conveniently divided into “macro” and “micro”, with macro goals commonly including systemic blood pressure, cardiac index, systemic venous oxygen saturation, and urinary output (Cheng et al. 2020). Microgoals may include lactate and/or lactate to pyruvate ratio, base deficit, oxygen consumption index, hemoglobin, and biomarkers (i.e., inflammatory, cardiac, and urinary).

A considerable lack of agreement exists regarding the general procedural and individual patient goals and their interplay, as well as the complex relationship between physiology, metabolism and coagulation. Similarly, there is a lack of understanding and agreement on causation and correlation of macro- and microgoals with metrics such as death, acute kidney injury, mechanical ventilation time, stroke, hemorrhage, transfusions, length of stay, readmissions, and costs (Brown et al. 2016; Speir et al. 2009). The association of acute kidney injury (AKI) and GDT is perhaps most well established and provides a good example of this complexity. AKI risk models commonly include age, female sex, impaired ventricular function, diabetes mellitus, chronic kidney disease, systemic hypertension, anemia, emergency status, and pre-operative intra-aortic balloon pump use, but fail to include the above modifiable macro- and microgoals (Lobdell et al. 2020; Meersch et al. 2017; Rubino et al. 2015). In large part, these models have tended to be pre-operative in nature, but it is the modifiable nature of GDT which increases its importance in terms of patient management standardization.

In addition to the aforementioned patient specific issues, operational details such as clinician education and experience, patient-staffing ratios, teamwork, pathways, protocols, and order sets may significantly influence the feasibility of employing and monitoring various approaches to GDT (Lobo et al. 2013). Data collection into electronic health records, and their lack of interoperability with registries as well as analytic limitations, also constrain our development of insight into GDT and compliance with established protocols designed to mitigate risks (Smirk et al. 2018). Similar limitations impede the incorporation of new technologies such as biomarkers into operational routines and risk assessment models (Obradovic et al. 2020). Finally, the duration of follow-up and definitions of specific metrics such as mortality, readmission, acute, and chronic kidney disease present challenges to a comprehensive and accurate assessment of the value of GDT.

### Acute kidney injury

Despite advances in the understanding of the cellular and molecular mechanisms of cardiac surgery-associated acute kidney injury (CSA-AKI) in the past decade (Bellomo et al. 2008), there is still uncertainty and a lack of consensus with regard to optimal prevention, early detection and management strategies. The high incidence of CSA-AKI and substantial impact on long-term patient outcomes as well as index admission mortality and hospital cost should drive our research efforts for improvement (Chen et al. 2021).

The current definition of AKI as described by the well-defined Kidney Disease: Improving Global Outcomes (KDIGO) criteria is neither sensitive nor specific for early identification of AKI. The limitations of serum creatinine and urine output have warranted the development of novel urinary biomarkers that have shown predictive value. Biomarkers allow the diagnosis of kidney injury to be made earlier, even in the absence of concurrent or subsequent kidney dysfunction (Meersch et al. 2017).

The most heavily studied biomarkers, NGAL and TIMP-2-IGFBP7, have demonstrated excellent performance in predicting CSA-AKI (Engelman et al. 2020; Meersch et al. 2014) and have the best accuracy and stability even in patients with congestive heart failure, diabetes, and chronic kidney disease (Kashani et al. 2013; Wang et al. 2017; Cruz et al. 2010). Controversies continue, however, regarding cutoff and test time as results from multiple studies have been inconsistent (Fan et al. 2019). A number of other biomarkers of cardiac function, inflammation and impaired renal function (Cystatin C, kidney injury molecule 1 (KIM-1), liver-type fatty acid-binding protein (L-FABP), uromodulin, neutrophil/(lymphocyte × platelet) ratio, urinary albumin, creatinine kinase-MB, NT-proBNP, interleukin 18 (IL-18), and fibroblast growth factor 23) have been identified in the past decade that show potential for characterizing the pathophysiology of disease and improving risk stratification (Wu et al. 2019a; Kim et al. 2015; Vives et al. 2019). The increased cost and lack of current evidence in the improvement of clinical outcomes has limited their utilization as we await results from large multicenter trials.

Though there is still a paucity of evidence, improvement in risk-stratification may encourage post-operative monitoring with biomarkers, early initiation of therapy, application of anti-inflammatory therapy and avoidance of nephrotoxic agents in high-risk patients (Kim et al. 2015). Studies such as the PrevAKI RCT have shown that implementation of a KDIGO bundle (supportive measures) in high risk patients before, rather than after a change in kidney function as identified by biomarkers, reduced the frequency and severity of CSA-AKI but there was no impact on secondary outcomes up to 90 days (Meersch et al. 2017). Future studies are needed to recognize the impact on long-term outcomes. Unfortunately, despite the known benefits, compliance with all 6 components of the KDIGO guidelines in clinical practice appears to be very low (Kullmar et al. 2020).

A number of potential pharmacologic and non-pharmacologic strategies have been described for the prevention and treatment of CSA-AKI. Agents including levosimendan, N-acetylcysteine, atrial natriuretic peptide agonists, fenoldapam, and dopamine have all failed to consistently demonstrate any benefit and perhaps may lead to potential harm (Nadim et al. 2018). Nevertheless, there are ongoing studies with some of these medications including novel agents (ularitide, EA-230), well known anesthestic drugs (dexmedetomidine and propofol), as well as amino acid therapies. Though previous studies have shown no benefit in the prevention of CSA-AKI with levosimendan, the phase 4 “Effects of Levosimendan in Acute Kidney Injury After Cardiac Surgery Trial” (LEVOAKI) is currently underway (Cote et al. 2020). Ularitide, an atrial natriuretic peptide agonist and EA-230, developed to replicate the immunomodulatory effects of human chorionic gonadotropin also have RCTs underway (TRUST, 2018-004871-11 and NCT 03145220, respectively) (Cote et al. 2020). Studies on amino acid administration through either intravenous infusion or via protein meals have shown an increase in renal plasma flow and an increase in GFR as well as faster recovery from severe acute renal failure. Currently, a phase 3 RCT with 3500 patients undergoing cardiac surgery is underway to test the hypothesis of kidney protection by amino acid infusion (NCT03709264) (Vives et al. 2019).

Dexmedetomidine, an alpha-2 adrenoreceptor agonist, has shown a reno-protective effect in several studies (Peng et al. 2020). A meta-analysis focusing on CSA-AKI prevention concluded that dexmedetomidine might reduce the incidence of AKI compared to placebo, but time and dosage must be further evaluated (Liu et al. 2018). Another recent systematic review and meta-analysis evaluating the effects of propofol versus volatile anesthetics on mortality and major clinical events in cardiac surgery patients revealed that the incidence of AKI and RRT was similar between the two, and that volatile anesthetics offered no renal protection (Bonanni et al. 2020). These results are conflicting with a previous report that describes significant renal protection in cardiac surgery patients with the use of volatile agents versus total intravenous anesthesia (Cai et al. 2014). In another study, the use of remote ischemic-preconditioning (RIPC) was associated with a reduced incidence of AKI with volatile anesthetics instead of propofol (Deferrari et al. 2018). The timing of renal replacement therapy remains controversial. The highly anticipated Artificial Kidney Initiation in Kidney Injury-2 (AKIKI-2) (Gameiro et al. 2020) and Standard Versus Accelerated Initiation of Dialysis in Acute Kidney Injury (STARRT-AKI) trials are now shedding some light on the previously conflicting results (Wald et al. 2020). While questions regarding optimal risk stratification and management, diagnosis, prevention, and treatment of CSA-AKI are ongoing, research efforts strive to make a positive impact on the detrimental health and cost effects related to this disease.

### Opioid analgesic reduction

Cardiac surgery has a longstanding relationship with the perioperative use of opioids. Dating back to the late 1960s opioid-based cardiac anesthesia (as described by Lowenstein (Lowenstein et al. 1969)) involved the use of large doses of intravenous morphine as the primary anesthetic for patients undergoing open aortic valve surgery. Although somewhat more refined, with providers opting for shorter-acting synthetic alternatives such as fentanyl and sufentanil, present-day cardiac anesthesia and analgesia still relies heavily on the administration of opioids (Silbert et al. 2006). Despite awareness of the harmful effects of perioperative opioid use, knowledge translation of the contribution of cardiac surgery to the opioid crisis to the medical community has been slow to gain momentum. Opioid-related side effects, including nausea, vomiting, constipation, and somnolence are recognized easily in low-to-moderate risk gastrointestinal, orthopedic and ambulatory surgeries, where recovery time is often directly associated with their occurrence (Dasinger et al. 2019; Tolvi et al. 2020). Similar issues are rarely prioritized—and are therefore underreported—in the context of cardiac surgery. Research has been devoted to establishing the association between certain surgical subtypes and new persistent opioid use (POU), defined as opioid use greater than 90 days after surgery in previously opioid-naïve patients (Brummett et al. 2017; Kent et al. 2019). The relationship has only recently been identified in cardiac surgery, where 6–15% of patients who undergo cardiac surgery develop POU, an association strengthened when the amount of postoperative opioid prescribed is high (Brown et al. 2020; Brescia et al. 2019; Clement et al. 2020).

Consensus guidelines have advocated for opioid reduction across numerous service lines (Gustafsson et al. 2019; Wu et al. 2019b), including a recent recommendation from the ERAS® Cardiac Society promoting the application of a “perioperative, multimodal, opioid-sparing, pain management plan” (Engelman et al. 2019). Unfortunately, there is relatively little evidence for non-opioid use in cardiac surgery and simple extrapolation of existing data in non-cardiac surgery may be inappropriate. Non-steroidal anti-inflammatory agents (NSAIDs) and COX-2 inhibitors are problematic due to an existing “black-box” warning in patients undergoing a CABG procedure and the high proportion of acute kidney injury in this population (US Food and Drug Administration 2020; Shaw 2017). A recent meta-analysis of gabapentinoids suggests the risk of somnolence may not outweigh any minor analgesic benefit (Verret et al. 2020). A landmark article investigating ketamine in cardiac surgery showed increased hallucinations without impacting opioid administration (Avidan et al. 2017). Neuraxial analgesia is rarely employed due to the use of systemic anticoagulation and studies of other forms of regional analgesia (i.e., erector spinae, paravertebral, sternal fascial plane block) have been limited (Mittnacht et al. 2019). Local anesthetic administration (intravenous or perineural) is both similarly underrepresented in the literature and complicated by a potentially increased incidence of perioperative arrhythmias. There is an emerging concept that may be thought of as “opioid sparing opioid therapy”, in which the use of a long-acting opioid such as methadone may lead to analgesic benefit in the first 30 days following cardiac surgery (Murphy et al. 2020). Finally, with rare exception, comprehensive phase-of-care-specific strategies have not been published, which provides little guidance to providers on how to construct a formal multimodal analgesic program (Williams et al. 2019; Grant et al. 2020). In the event non-opioid strategies are employed, additional systems-level issues have not been addressed. Prior examples of an opioid free analgesic program associated with an ERAS→ program in colorectal surgery failed to reduce opioids on discharge (Brandal et al. 2017), which is likely to be repeated in cardiac surgery unless meaningful analgesic prescription planning is addressed. Despite a longstanding relationship with opioids, the cardiac surgical community has lagged behind other disciplines in recognition of the issues associated with opioid use and the development of systematic strategies to uniquely address opioid-based anesthesia and analgesia in the cardiac surgical population.

### Delirium

Delirium is a serious perioperative complication in which patients present with acute alterations in levels of consciousness, attention, and disordered cognition (Association AP 2013). Delirium occurs commonly following cardiac surgery patients with contemporary studies indicating a prevalence of at least 1 in 5 patients following their procedure (Jung et al. 2015; Arenson et al. 2013). The “3-strike” paradigm denotes the key determinants that can lead to an increased risk of delirium in patients undergoing cardiac surgery: (1) a baseline vulnerability of the brain in the older adult cardiac patient that results in a lower bio-psycho-social reserve; (2) suffering an acute cardiac stressor (i.e., new event such as cardiac surgery); and (3) further compounding post-event stressors related to processes of care in the ICU and ward phases of care (Arora et al. 2017). Importantly, the occurrence of postoperative delirium in the cardiac surgery patient has been shown to be associated with multiple short- and long-term adverse outcomes, including increased mortality, loss of functional independence and greater prevalence of long-term cognitive decline. Further work must include a greater awareness for the cardiac surgeon and perioperative specialist of the impact of delirium on the post-procedure cardiac patient. There are three key considerations for the perioperative team’s management of this vexing complication (Sanjanwala et al. 2020; Dubiel et al. 2020), and these are discussed in the following paragraphs.

The cardiac perioperative team frequently has an opportunity to obtain information about patients before their procedure. As such, an important step in the prevention of delirium is the identification of baseline risk. This should include an assessment of frailty and cognition (such as the Montreal Cognitive Assessment (Nasreddine et al. 2005) or Mini-Cog (Borson et al. 2000)) as well as anxiety, depression, and pre-procedure pain (Kosar et al. 2014). This information serves to alert the team of increased risk of postoperative delirium and to identify possible targets to optimize prior to their procedure (for example prehabilitation (Arora et al. 2018)). In addition, identification of increased risk of delirium should be included in the communication of information during clinical handoffs to ensure the perioperative team remains vigilant throughout the patient journey (Chatterjee et al. 2019).

In the postoperative phase, the variable manifestations of delirium symptom presentation, in conjunction with its fluctuating course, pose challenges for the bedside practitioner to diagnose in clinical practice (Neufeld et al. 2013). Consequently, delirium is often under-recognized, with rates of missed diagnosis reported in greater than two-thirds of cases (Neto et al. 2012). Therefore, the use of a dedicated screening tool, appropriate to the clinical environment, is essential to detect delirium when it occurs at the earliest time point in order to permit the perioperative team to determine the potential etiology (Devlin et al. 2018). While there is no universally accepted delirium screening tool for the ICU, the confusion assessment method for the intensive care unit (CAM-ICU (Ely et al. 2001)) or the intensive care delirium screening checklist (ICDSC (Bergeron et al. 2001)) are the most commonly referenced tools. Bedside screening for delirium should occur regularly, ideally once per nurse shift (Engelman et al. 2019). For the ward, it would be reasonable for the perioperative team to consider incorporating tools that are more brief (and therefore clinically feasible) such as the 3-min version of the confusion assessment method (3D-CAM (Marcantonio et al. 2014)) or the “4 A’s Test” (4AT (Tieges et al. 2020)); however, both require additional validation in the cardiac surgery patient.

Owing to the complexity of delirium pathogenesis, a single intervention will unlikely be effective once delirium occurs (Maldonado 2013). The primary approach for delirium management in the cardiac surgery patient, following the use of routine delirium risk identification and screening, is reducing risk through patient optimization and mitigation of iatrogenic pathology. In the preoperative setting, two key geriatric syndromes that impact postoperative outcomes, including delirium, are frailty and malnutrition. Previous investigations have identified that approximately 50% of older adults undergoing cardiac surgery can be considered frail (Jung et al. 2015; Lytwyn et al. 2017) as defined by a cumulative decline in multiple physiological systems resulting in a vulnerability to stressor events (Rockwood and Mitnitski 2007; Fried et al. 2001). This results in a population who are at a higher risk for poor outcomes, including delirium. This is further compounded by malnutrition, which is an unintentional nutritional imbalance, but not necessarily only decreased intake. It is present in 20% of patients presenting for cardiac surgery (Patel et al. 2014). Both of these syndromes, in addition to mental health issues, may be amenable to pre-operative optimization (i.e., prehabilitation) (Arora et al. 2018). Further studies are needed to determine the effectiveness of these interventions in preventing delirium vulnerability in older adult patients undergoing cardiac surgery.

It is increasingly recognized that the perioperative team itself can contribute to iatrogenic delirium risk (Vasilevskis et al. 2010; Vasunilashorn et al. 2018). In the postoperative phase of care, therefore, it is important to utilize an interdisciplinary approach to managing postoperative delirium. The principles of this approach are contained with the most current Society of Critical Care Medicine Pain, Agitation/Sedation, Delirium, Immobility (mobilization/rehabilitation), and Sleep (disruption) recommendations (Devlin et al. 2018). Specific to the preventative management of delirium, routine delirium screening, early mobilization and sleep promotion are the mainstays of this clinical pathway (Arora et al. 2017). In addition, the surgical and perioperative team should include consideration of less invasive procedures and development of strategies for minimizing opioid use for postoperative pain management (Engelman et al. 2019; Arora et al. 2017).

Contemporary societal recommendations have advocated against the routine use of benzodiazepines and antipsychotic agents in favor of non-pharmacologic strategies as the first-line components of management (Engelman et al. 2019; Arora et al. 2003). Furthermore, there is no evidence that prophylactic (Page et al. 2013) or routine (Girard et al. 2018) antipsychotic use reduces delirium and these should therefore be reserved for those patients with significantly distressing symptoms or those who pose a safety concern to themselves or others (Devlin et al. 2018). More research is needed surrounding potential pharmacologic treatments. First-line non-pharmacologic strategies are consistent with the preventative measures described earlier.

An aging population will continue to contribute to a greater prevalence of heart disease in an older and increasingly frail cardiac patient population, who are themselves vulnerable to delirium. The perioperative team should view the occurrence of postoperative delirium as a quality indicator for health care delivery because it is common, associated with iatrogenesis and integrally linked to processes of care (Fatehi Hassanabad et al. 2021). The contemporary cardiac team should seek to incorporate routine risk identification during the preoperative evaluation and consider opportunities to optimize vulnerable patients while they await surgery (Enomoto et al. 2021). Modifications of intraoperative and postoperative processes of care to mitigate risk of postoperative delirium need to be undertaken and contextualized to the providing center’s clinical environment (Peng et al. 2020; Siripoonyothai and Sindhvananda 2021). Lastly, we strongly encourage the development and facilitation of team-based, multicomponent strategies to mitigate the occurrence of delirium in the postoperative cardiac surgery patient.

## Conclusions

It is readily apparent that there is much work to be done if we are to improve value (better patient reported outcomes and decreased costs) in perioperative cardiac medicine. There are implications for resource utilization as well as clinical outcome improvements in the five clinical topics and research questions (see Additional file 1) we have outlined in this review. We hope that this represents the first step in identifying a research agenda that will increase consensus and reduce controversy by providing answers to well-articulated important clinical questions.

## Supplementary Information

**Additional file 1.** Research Questions.

## Data Availability

Not applicable

## Abbreviations

3D-CAM: 3-minute version of the confusion assessment method
4AT: 4 A’s test
AKI: Acute kidney injury
CABG: Coronary artery bypass grafting
CAM-ICU: Confusion assessment method for the intensive care unit
CSA-AKI: Cardiac surgery-associated acute kidney injury
ERAS: Enhanced recovery after surgery
ERAS-CS: Enhanced recovery after surgery–cardiac surgery
ESAs: Erythropoietin stimulating agents
GDT: Goal-directed therapy
ICDSC: Intensive care delirium screening checklist
IGFBP7: Insulin-like growth factor-binding protein 7
KDIGO: Kidney Disease: Improving Global Outcomes
KIM-1: Kidney injury molecule-1
L-FABP: Liver-type fatty acid-binding protein
NGAL: Neutrophil gelatinase-associated lipocalin
OPCAB: Off-pump coronary artery bypass grafting
PBM: Patient blood management
POQI: Perioperative quality initiative
POU: Persistent opioid use
RIPC: Remote ischemic preconditioning
TIMP-2: Tissue inhibitor of metalloproteinases 2
