# Supplementary material for: Controversies in enhanced recovery after cardiac surgery
Source: Perioper Med (Lond). 2022 Apr 28;11:19. doi: 10.1186/s13741-022-00250-7 (PMC9047268; doi:10.1186/s13741-022-00250-7)
Supplement: Supplementary file 1 — Additional file 1. Research Questions. [file 13741_2022_250_MOESM1_ESM.docx]

**Additional file 1 – Research Questions**

**Patient Blood Management**

*How and when should patients undergoing non-emergent cardiac surgery be evaluated and treated for preoperative anemia?*

*Notwithstanding the fact that the decision to transfuse red blood cells involves considering many factors, what is the appropriate hemoglobin concentration to trigger transfusion in cardiac surgery while on cardiopulmonary bypass, intraoperatively before and after revascularization, and postoperatively?*

*Should the non-pharmacologic, intraoperative blood conservation techniques of acute normovolemic hemodilution and autologous prime be used routinely to reduce red blood cell transfusion requirements in cardiac surgery?*

**Goal Directed Therapy**

*Does the method by which goal directed therapy is delivered to cardiac surgical patients have an effect on outcomes?*

*Do the individual goals in GDT matter, or is it the overall concept that makes a difference?*

*Are there aspects of GDT that are specific to cardiac surgical patients?*

**Acute Kidney Injury**

*In which cardiac surgery patient population is the utilization of biomarkers most cost-effective for predicting AKI? How can this be combined with renal protection strategies to offer optimal renal protection protocols or bundles?*

*Should renal replacement therapy be individualized to incorporate other factors than timing such as the cause of injury, speed of progression and other comorbidities?*

*How do anesthetic pharmacologic strategies including the use of propofol, dexmedetomidine or volatile agents impact the incidence of CSA-AKI?*

**Opioid Analgesic Reduction**

*Which non-opioid medications and techniques provide optimal analgesia, with opioid sparing and have optimal safety profiles for use in the cardiac surgical population?*

*How can opioid-sparing strategies employed in the perioperative setting be translated to meaningful reduction in intermediate and long-term opioid use upon discharge after cardiac surgery?*

*How does chronic opioid use (i.e., opioid tolerance, opioid abuse) impact pain management or dictate opioid administration in the cardiac surgical setting?*

**Delirium Research Qs**

*What is the role of specific sedative drugs in the development, prevention and management of delirium in the postoperative cardiac surgery patient?*

*What is the most valid and effective delirium screening tool in the post-operative cardiac surgery ward?*

*What non-drug treatments might reduce the incidence of postoperative delirium?*
